# Development and psychometric evaluation of the women shift workers’ reproductive health questionnaire: a sequential exploratory mixed-method study

**DOI:** 10.1186/s12978-020-00994-9

**Published:** 2020-09-29

**Authors:** Maryam Nikpour, Aram Tirgar, Fatemeh Ghaffari, Abbas Ebadi, Hamid Sharif Nia, Fatemeh Nasiri-Amiri

**Affiliations:** 1 PhD of Health Science, Non-Communicable Pediatric Disease Research Center, Health Research Institute, Babol University of Medical Sciences, Babol, I.R Iran; 2 PhD of Occupational Health, Professor of Social Determinants of Health Research Center, Health Research Institute, Babol University of Medical Sciences, Babol, I.R Postal Code: 47745-47176 Iran; 3 PhD of Nursing, Associated of Professor, Nursing Care Research Center, Health Research Institute, Babol University of Medical Sciences, Babol, I.R Iran; 4 Professor of Behavioral Sciences Research Center, Life style institute, Baqiyatallah University of Medical Sciences, Tehran, IR Iran; 5 Nursing Faculty, Baqiyatallah University of Medical Sciences, Tehran, IR Iran; 6 PhD of Nursing, Assistant Professor, School of Nursing and Midwifery Amol, Mazandaran University of Medical Sciences, Sari, Iran; 7 PhD of Reproductive Heath, Associated of Professor, Infertility and Health Reproductive Research Center, Health Research Institute, Babol University of Medical Sciences, Babol, Iran

**Keywords:** Reproductive health, Women shift workers, Instrument development, Psychometric evaluation

## Abstract

**Background:**

There is no standard and comprehensive questionnaire for reproductive health assessment among women shift workers. This study aimed at the development and psychometric evaluation of the Women Shift Workers’ Reproductive Health Questionnaire.

**Methods:**

This sequential exploratory mixed-method study was conducted in a qualitative (item generation) and a quantitative (psychometric evaluation) phase. In the qualitative phase, the primary item pool of the questionnaire was generated based on the findings of the qualitative content analysis of 21 interviews held with 21 women shift workers as well as the findings of a literature review. In the quantitative phase, the face, content, construct, convergent, and discriminant validity and the reliability of the questionnaire were assessed. For construct validity assessment through exploratory and confirmatory factor analyses, 620 women shift workers were conveniently selected to fill out the questionnaire. Reliability assessment was done through assessing internal consistency, stability, and composite reliability.

**Results:**

The primary item pool contained 88 items. During face and content validity, item number was reduced to 55. Construct validity assessment through factor analysis revealed that 56.50% of the total variance was explained by five factors with 34 items. The factors were named motherhood, general health, sexual relationships, menstruation, and delivery. Confirmatory factor analysis confirmed the fit of the five-factor model. The Cronbach’s alpha and the composite reliability value of the questionnaire were more than 0.7.

**Conclusion:**

The Women Shift Workers’ Reproductive Health Questionnaire is a valid and reliable instrument and can be used for reproductive health assessment among women shift workers.

## Plain English summary

Shift work, defined as working between 18:00 and 07:00, has significant effects on different aspects of reproductive health, including reproductive system, menstruation, sexual relationships, pregnancy outcomes, and premenopausal symptoms. There is no standard and comprehensive questionnaire for reproductive health assessment among women shift workers. Therefore, this study was conducted to develop the Women Shift Workers’ Reproductive Health Questionnaire and evaluate its psychometric properties.

This study was conducted in two phases. In the first phase, a questionnaire was developed through interviewing 21 women shift workers selected from round-the-clock centers (including hospitals, welfare and rehabilitation centers, and factories) in Mazandaran province, Iran. In the second phase, twelve experts (in midwifery, gynecology and obstetrics, and occupational health) commented on the grammar, wording, allocation, and scoring of the questionnaire items. The questionnaire was revised based on their comments. The final questionnaire contained 34 items in five dimensions, namely motherhood, general health, sexual relationships, menstruation, and delivery. The Women Shift Workers’ Reproductive Health Questionnaire is a valid and reliable questionnaire for assessing women shift workers’ reproductive health.

## Introduction

Women’s reproductive health has a broad scope and encompasses all sensitive stages of life from birth to menopause [1]. A wide range of physical, mental, social, and environmental factors can affect reproductive health. Shift work, defined as working between 18:00 and 07:00, is one of these factors [2, 3]. More than two third of women workers are in reproductive age [4] and most women workers in service jobs, particularly in hospital environments [5], are shift workers.

Shift work affects different aspects of reproductive health, including reproductive system, menstruation [6], sexual relationships [7], pregnancy outcomes [8, 9], and premenopausal symptoms [10]. It alters circadian rhythm, reduces the level of melatonin hormone [11], alters the production of sex hormones, and thereby, endangers reproductive health [12]. Moreover, work in holidays, long working hours, sleeplessness, and chronic fatigue associated with shift work can damage women’s reproductive health [13].

Reproductive health assessment is an essential prerequisite to the development and use of interventions for its promotion. Such assessment necessitates valid and reliable culturally-appropriate instruments. There are several instruments for reproductive health assessment. For instance, the Survey of Shift Questionnaire is a standard instrument which assesses the effects of shift work on physical and mental health and personal, familial, and social relationships [14]. Some other instruments in this area include Reproductive Health Assessment Toolkit for Conflict-Affected Women [15] and Sexual and Reproductive Health Needs Assessment among Mobile and Vulnerable Population [16]. None of the available reproductive health assessment instruments is specific to shift workers and hence, most previous studies into women shift workers’ reproductive health focused on the assessment of some of its aspects such as sexual function [7], pregnancy outcomes [9], menstruation [17], and infertility [18]. Thus, developing a specific instrument for assessing women shift workers’ reproductive health seems necessary. The present study was conducted to address this gap. The aim of the study was to develop the Women Shift Workers’ Reproductive Health Questionnaire (WSW-RHQ) and evaluate its psychometric properties.

## Methods

This sequential exploratory mixed-method study was conducted in a qualitative and a quantitative phase. The methods of this study were published in detail elsewhere as a protocol study [4].

### The qualitative phase

The aim of this phase was to explore the concept of women shift workers’ reproductive health and its dimensions for the generation of the WSW-RHQ primary item pool. Participants were 21 women shift workers recruited from round-the-clock centers (including hospitals, nursing homes, welfare and rehabilitation centers, and factories) in Qaemshahr, Amol, and Babol, Mazandaran province, Iran. Women shift workers were included in the study if they were married, aged 18–45, had experienced pregnancy and breastfeeding, and had a work experience of more than 2 years. Sampling was purposively performed with maximum variation respecting participants’ age, work experience, educational level, financial status, number of children, and occupation.

Data were collected via semi-structured interviews held according to participants’ preferences in a private room at their workplaces. Examples of interview questions were, “In your opinion, what are the effects of shift work on reproductive health?” “What factors affect reproductive health?” “What were the effects of shift work on your pregnancy or breastfeeding?” “What have been the effects of shift work on your sexual behaviors?” In order to collect more in-depth data, we also used probing questions such as, “Can you explain more about this?” “Can you provide an example?” At the end of each interview, the interviewee was asked if she wanted to add anything else about shift work and reproductive health. Sampling and data collection were kept on up to data saturation which was achieved after 21 interviews with 21 women shift workers. Data saturation is the point at which no new data are obtained from the interviews. Interviews lasted 25–70 min.

Data were analyzed through the conventional content analysis method recommended by Graneheim and Lundman [19]. During content analysis, the dimensions and the components of women shift workers’ reproductive health were identified. Data trustworthiness was ensured using the four criteria proposed by Guba and Lincoln, namely credibility, dependability, confirmability, and transferability [20].

An item pool was generated based on the dimensions and the components of women shift workers’ reproductive health identified during conventional content analysis. Moreover, a review of the existing literature and reproductive health assessment instruments was performed and its findings were used for item generation.

### The quantitative phase

In this phase, we assessed the psychometric properties of WSW-RHQ, namely its face, content, construct, convergent, and discriminant validity as well as its reliability. Reliability assessment was done through internal consistency, stability, and composite reliability assessments.

#### Face validity assessment

Face validity was assessed using qualitative and quantitative methods. In the qualitative method, ten women shift workers were interviewed about the difficulty, appropriateness, and ambiguities of the items and then, the items were revised according to their comments. Then, quantitative content validity assessment was performed through calculating item impact score. Accordingly, ten women shift workers were asked to rate the importance of each item on a five-point scale from 1 (“The lowest importance”) to 5 (“The highest importance”). Item impact score was calculated by multiplying the mean item importance score by the number of women shift workers who rated the importance of that item 4 or 5 [21].

#### Content validity assessment

Content validity was also assessed using qualitative and quantitative methods. For qualitative content validity assessment, twelve experts (in reproductive health, midwifery, gynecology and obstetrics, and occupational health) were invited to read WSW-RHQ and comment on the grammar, working, item allocation, and scoring of its items. Items were revised based on their comments. Quantitative content validity assessment was performed through calculating content validity ratio (CVR) and content validity index (CVI). For CVR calculation, ten of the above-mentioned twelve experts were asked to rate the essentiality of each item. An item CVR of 0.64 or more was considered acceptable [22]. Moreover, items which were considered essential by nine experts were acceptable [23]. For CVI calculation, the same experts were asked to rate the relevance of each item. An item CVI of 0.78 or more was considered acceptable [24].

#### Primary reliability assessment

Before construct validity assessment, a pilot study was carried out to assess the primary reliability of WSW-RHQ. Accordingly, fifty women shift workers completed the questionnaire. The Cronbach’s alpha of the questionnaire was 0.92 and none of the items had an inter-item correlation coefficient of less than 0.3.

#### Construct validity assessment

Construct validity was assessed via exploratory and confirmatory factor analyses. Based on the rule of thumb, the sample size was determined to be 300 [25]. Accordingly, two samples (620 participants in total) were conveniently selected for exploratory and confirmatory factor analyses. In exploratory factor analysis, latent factors were extracted through maximum likelihood estimation with equimax rotation and Horn’s parallel analysis. Kaiser-Meyer-Olkin (KMO) measure of sampling adequacy and the Bartlett’s test were used. A KMO value of 0.8 or more was considered acceptable [26]. The minimum acceptable factor loading was 0.3 [27]. After exploratory factor analysis, confirmatory factor analysis was used to confirm the factor structure model extracted in exploratory factor analysis. Indices for model goodness of fit assessment were root mean score error of approximation (RMSEA), comparative fit index (CFI), parsimony comparative fit index (PCFI), goodness of fit index (GFI), adjusted goodness of fit index (AGFI), minimum discrepancy function divided by degrees of freedom (CMIN/DF), normed fit index (NFI), and parsimony normal fit index (PNFI).

#### Normal distribution, outliers, and missing data

The normality of univariate and multivariate data was assessed through assessing skewness (±3) and kurtosis (±7). Multivariate outliers were assessed through the Mahalanobis squared distance (*P* < 0.001) and multivariate normality was assessed using the Mardia coefficient of multivariate kurtosis (> 20) [28]. The distribution of missing data was also assessed using multiple imputation and then, missing values were replaced by the mean score of participants’ responses .

#### Convergent and discriminant validity assessment

Convergent and discriminant validity were assessed using the Fornell and Larcker method. Accordingly, the average variance extracted (AVE), maximum shared variance (MSV), and composite reliability (CR) were assessed [29]. Convergent validity is confirmed when AVE is greater than 0.5 and CR is greater than AVE, while discriminant validity is confirmed when AVE is greater than MSV [30].

#### Reliability assessment

For internal consistency assessment, we calculated Cronbach’s alpha, McDonald omega, and average inter-item correlation [31]. Satisfactory internal consistency is established when Cronbach’s alpha value is greater than 0.70 [32] and average inter-item correlation coefficient is 0.2–0.4. For test-retest stability assessment, twenty women shift workers were asked to twice complete WSW-RHQ. Then, intraclass correlation coefficient (ICC) was calculated using the two-way mixed effects model and the absolute agreement method. After that, CR was calculated and a CR value of more than 0.7 was considered as acceptable reliability [33].

#### Absolute reliability

As ICC does provide information about the accuracy of the scores, absolute reliability was calculated using the standard error of measurement (SEM) and the following formula [34], $SEM= pooled\  SD\sqrt{1- ICC}$.

#### Simplicity of using WSW-RHQ

Simplicity of using WSW-RHQ was assessed based on the average time needed for its completion and the percentage of participants who did not answer each item [35]. To determine the average time needed for WSW-RHQ completion, the time of WSW-RHQ completion among the first fifty participants was measured and averaged. Non-response rate was calculated using the data obtained from all participants.

#### Floor and ceiling effects

Floor and ceiling effects exist when more than 15% of participants obtain respectively the lowest and the highest possible total score of the intended instrument [36]. These effects were also assessed using the data obtained from all participants.

#### Scoring

WSW-RHQ items were scored on a Likert scale from 1 to 5. Then, the total scores of WSW-RHQ and its dimensions were changed into a 1–100 scale using the following formula [34],

$$ Total\kern0.5em score=\frac{Obtained\kern0.5em crude\kern0.5em score- The\kern0.5em lowest\kern0.5em crude\kern0.5em score}{The\kern0.5em highest\kern0.5em crude s\kern0.5em core- The\kern0.5em lowest\kern0.5em crude\kern0.5em score}\times 100 $$

#### Data analysis

Statistical data analysis was done using the SPSS-AMOS24. Horn’s parallel analysis was done using the SPSS R-menu v2.

### Ethical considerations

This study was approved by the Ethics Committee of Babol University of Medical Sciences, Babol, Iran (code: MUBABOL.HRI.REC.1395.58). Informed consent was obtained from all participants and they were informed about the confidential management of the study data.

## Results

### Item generation

The primary item pool, generated based on the findings of the qualitative phase, included 85 items. Three more items were added to the item pool based on the findings of the literature review. Of course, all these three items were excluded during psychometric evaluation. Finally, 88 items were subjected to psychometric evaluation.

### Face and content validity

Five items were revised based on participants’ comments in qualitative face validity assessment and no item was deleted in quantitative face validity assessment. In qualitative content validity assessment, sixteen items were merged into eight items and hence, the number of items reduced to eighty. In quantitative content validity assessment, eighteen items were deleted due to low CVR and seven items were deleted due to low CVI. Finally, 55 items remained in the questionnaire for construct validity assessment.

### Construct validity assessment

For construct validity assessment, 620 women shift workers filled out WSW-RHQ (410 participants for exploratory factor analysis and 210 participants for confirmatory factor analysis). In total, 37 questionnaires were excluded due to incomplete answering and data analysis was performed on the data obtained from 583 participants (response rate = 94%). The means of participants’ age and work experience were 35.46 ± 5.40 and 11.75 ± 5.89, respectively. Most participants had university degrees (76%) and lived in urban areas (84%). Moreover, almost half of them had one child (50.4%) and 13% of them had the experience of one abortion (Table 1).

**Table 1:** Participants’ demographic characteristics

Characteristics		Mean ± SD	Total
Age (Years)		35.46 ± 5.40	583
Work experience (Years)		11.78 ± 5.89	583
Menarche age (Years)		13.23 ± 1.46	529
Characteristics		N (%)	Total
Educational status	Secondary	66 (11.30)	583
	Diploma	74 (12.70)	
	Bachelor’s	443 (76.00)	
Place of residence	Urban areas	490 (84.00)	583
	Rural areas	93 (16.00)	
Income level	Sufficient	210 (36.00)	583
	Moderately sufficient	283 (48.56)	
	Insufficient	90 (15.44)	
Occupation	Healthcare provider	423 (14.4)	583
	Mother aid or nurse aid	84 (72.6)	
	Laborer	43 (27.4)	
	Service worker	33 (5.7)	
Menarche age (Years)	13	322 (60.9)	529
	> 13	207 (39.1)	
Number of children	1	276 (50.4)	548
	2	272 (49.6)	
Number of abortions	0	482 (87.8)	549
	1	67 (12.2)	
Route of delivery	Normal vaginal	160 (30)	534
	Cesarean section	339 (63.4)	
	Both (in different deliveries)	35 (6.6)	

**Fig. 1:**
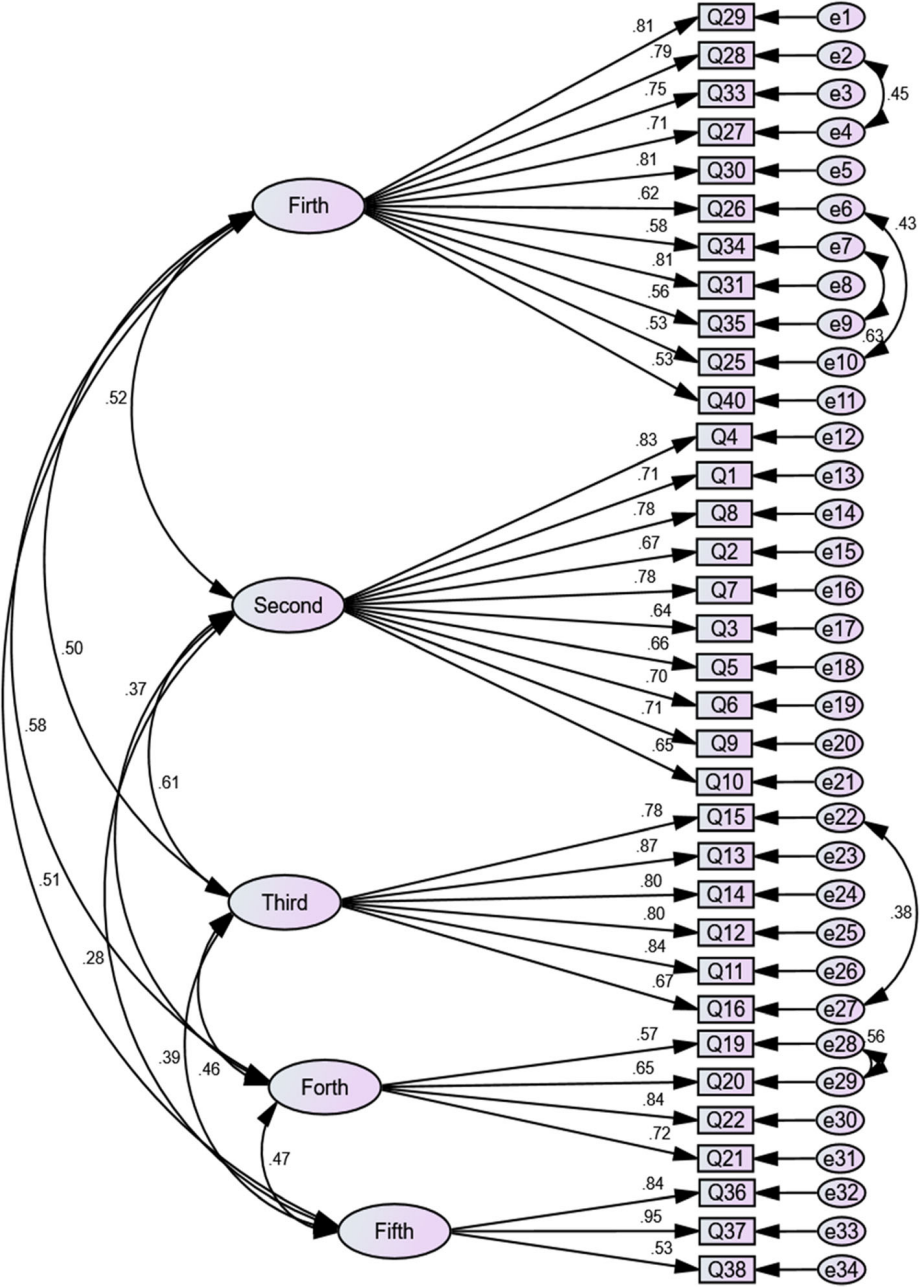
The confirmatory factor analysis model of WSW-RHQ

The KMO measure was equal to 0.935 and the Bartlett test was statistically significant (Chi-square value = 7427.74; *P* < 0.001). Five factors were extracted in exploratory factor analysis with parallel analysis which explained 56.50% of the total variance (Table 2). These five factors included 34 items and were named motherhood, general health, sexual relationships, menstruation, and delivery. In confirmatory factor analysis, after correcting the model and determining the correlation among measurement errors (Fig. 1), the Chi-square GFI was calculated to be 82.93 (*P* < 0.001). Then, other goodness of fit indices were determined to be as the following: PCFI = 0.801; PNFI = 0.718; CMIN/DF = 2.030; RMSEA = 0.720; GFI = 0.973; AGFI = 0.836; CFI = 0.977. All these indices confirmed model fit (Table 3). Significant correlations were observed between items 15 and 16, 19 and 20, 25 and 26, 27 and 28, and 34 and 35 (Fig. 1).

**Table 2:** The explained variances and eigenvalues of the WSW-RHQ dimensions and the factor loadings and the communality values of their items

Items		Factor loading	Item communality	Variance (%)	Eigenvalue
1	29. During pregnancy, I had a poor nutrition due to consuming workplace foods.	0.80	0.68	22.29	7.58
	28. During pregnancy, I easily got angry due to my shift work..	0.74	0.60		
	33. My insufficient sleep during breastfeeding was due to shift work..	0.74	0.56		
	27. My anxiety and apprehension during pregnancy was due to work conditions.	0.73	0.57		
	30. During pregnancy, I couldn’t prepare healthy food due to fatigue and lack of time.	0.72	0.62		
	26. Working during pregnancy exacerbated my pelvic pains.	0.72	0.52		
	34. When I returned to work, I had breast engorgement and pain.	0.71	0.45		
	31. During pregnancy, I could not rest due to my shift work.	0.70	0.60		
	35. When I returned to work, breastfeeding turned into a concern.	0.70	0.41		
	25. Work conditions exacerbated my nausea and vomiting during pregnancy.	0.65	0.41		
	40. Because of my shift work, I had to prematurely wean my baby.	0.41	0.31		
2	4. My feeling of early aging is due to shift work.	0.87	0.70	14.29	4.94
	1. My physical fatigue is due to work conditions.	0.81	0.57		
	8. Because of my shift work, I don’t have adequate time for satisfying my womanly needs (such as going to beauty shop, grooming, etc.).	0.78	0.58		
	2. My insufficient sleep is due to shift work. .	0.72	0.56		
	7. My aggression is due to shift work..	0.70	0.53		
	Shift work reduced my resistance to illnesses.	0.67	0.55		
	5. My stress is due my work conditions. .	0.67	0.46		
	6. I have not enough happiness due to my work conditions.	0.70	0.50		
	9. Because of my shift work, I don’t have adequate time for screening tests such as Pap smear and breast examination.	0.60	0.46		
	10. Because of my shift work, I don’t have adequate time for pleasurable activities such as sport, recreation, and travel.	0.55	0.40		
3	15. I escape from sexual relationships or reluctantly engage in it.	0.90	0.70	10.43	3.54
	13. My sexual pleasure reduced due to my work-related fatigue.	0.67	0.71		
	14. I don’t reach sexual climax due to my work-related fatigue.	0.77	0.63		
	12. My libido reduced due to work-related fatigue and insufficient sleep.	0.76	0.70		
	11. My work-related fatigue has caused me not to positively respond to my husband’s request for sex.	0.73	0.70		
	16. My reduced interest in sexual relationships has reduced the intimacy between me and my husband.	0.63	0.47		
4	19. During menstruation, I feel pain in my back and lower abdomen if I am at work. .	0.85	0.70	5.91	2.01
	20. During menstruation, I need analgesics to reduce my pain if I am at work.	0.85	0.65		
	My work aggravates premenstrual physical symptoms (such as headache, breast tenderness, and weakness).	58.0	52.0		
	21. I suffer from irregular menstruation due to my shift work.	0.49	0.38		
5	36. I had a premature delivery due to job strain. .	0.96	0.78	5.67	1.93
	37. My premature labor pain was due to shift work.	0.89	0.72		
	38. My spotting during pregnancy was due to my work conditions.	0.48	0.42

**Table 3:** Goodness of fit indices in confirmatory factor analysis

Indices Model	χ^**2**^	df	***P*** value	CMIN/DF	RMSEA	PCFI	PNFI	AGFI	GFI	CFI
Corrected	82.934	39	0.000	2.30	0.072	0.801	0.718	0.836	0.973	0.977

*RMSEA* Root Mean Score Error of Approximation (RMSEA), *CFI* Comparative Fit Index, *PCFI* Parsimony Comparative Fit Index, *GFI* Goodness of Fit Index, *AGFI* Adjusted Goodness of Fit Index, *CMIN/DF* Minimum Discrepancy Function Divided by Degrees of Freedom, *NFI* Normed Fit Index, *PNFI* Parsimony Normal Fit Index

### Reliability assessment

The Cronbach’s alpha values of WSW-RHQ dimensions were 0.82–0.92 and test-retest ICC was 0.97. SEM was ±2.14 and inter-item correlation coefficient was more than 0.4. Table 4 shows McDonald omega and ICC values.

**Table 4:** The convergent and discriminant validity, internal consistency, and stability of WSW-RHQ

Indices Factors	MSV	AVE	CR	α (CI 95%)	AIC	Ω	ICC
Motherhood	0.33	0.57	0.90	0.91 (0.90–0.92)	0.49	0.91	0.97
General Health	0.37	0.51	0.91	0.91 (0.90–0.92)	0.51	0.91	0.91
Sexual relationships	0.37	0.63	0.91	0.90 (0.89–0.91)	0.62	0.91	0.92
Menstruation	0.33	0.59	0.79	0.82 (0.77–0.83)	0.51	0.82	0.86
Delivery	0.26	0.63	0.82	0.83 (0.80–0.85)	0.62	0.86	0.88

α: Cronbach’s alpha; Ω: McDonald’s Omega; *CR* Composite reliability, *AVE* Average Variance Extracted, *MSV* Maximum Shared Squared Variance, *ASV* Average Shared Squared Variance

### Simplicity of using WSW-RHQ

Average time for filling out the questionnaire was 10 min in the range of 5–20. Except for the item 29, the non-response rates of the other items were 0–2.99%. The non-response rate of the item 29 was 4%.

### Floor and ceiling effects

The percentages of participants who obtained the lowest and the highest possible scores of WSW-RHQ and its dimensions were less than 15%.

### WSW-RHQ scoring

WSW-RHQ included 34 items in the five dimensions of motherhood (eleven items), general health (ten items), sexual relationships (six items), menstruation (four items), and delivery (three items). If all items are equally weighted 1, item scores can be changed into the 0–100 scale using the following formula, *Total FSWRHQ score* = ((*Crude score* − 34)/136) × 100. Lower WSW-RHQ scores show better reproductive health status among women shift workers.

## Discussion

The aim of this study was to develop WSW-RHQ and evaluate its psychometric properties. Findings showed that the five factors extracted from the questionnaire explained 55.60% of the total variance of its total score, denoting its appropriateness for measuring reproductive health among women shift workers. When the amount of the explained variance is more than 50%, factor extraction is considered appropriate [27].

The first dimension of WSW-RHQ was named motherhood and contained eleven items (i.e. almost around one third of all items). This dimension explained 22.29% of the total variance. The items of this dimension were related to the outcomes of pregnancy and breastfeeding. An explanation for the high number of items in this dimension is the great importance of pregnancy and breastfeeding for women shift workers so that some participants even equated reproductive health with prenatal health. The importance of pregnancy for Iranian women has also been confirmed in other studies [37, 38]. The other explanation is that the greatest effects of shift work on reproductive health might have been on pregnancy and breastfeeding, as confirmed by some earlier studies [39, 40]. The World Health Organization names perinatal health as safe motherhood, considers it as one of the twelve dimensions of reproductive health, and highlights that perinatal care is among reproductive health rights (https://www.who.int/westernpacific/health-topics/reproductive-health). The Sexual and Reproductive Health Needs Assessment among Mobile and Vulnerable Population instrument also contains items on breastfeeding. The number of breastfeeding-related items in that instrument is two out of 114 items (1.75%) [16].

General health, the second dimension of WSW-RHQ, explained 14.29% of the total variance. Our participants considered physical and mental health as the main part of their reproductive health because the items of this dimension constituted almost 29% of all WSW-RHQ items. Lebanese women in a qualitative study also reported general and mental health as one of the main aspects of reproductive health [41]. Similarly, the World Health Organization defines reproductive health as physical, mental, and social well-being in relation to reproduction [1]. According to this definition, any physical or mental problem which causes alterations in the reproductive system can be considered as a component of reproductive health.

The third dimension of WSW-RHQ was related to sexual relationships. This dimension included six items on the quantity and the quality of sexual relationships and explained 10.43% of the total variance. The World Health Organization introduced sexual health as a component of reproductive health [42]. It is among the basic needs for achieving the goals of development in the third millennium [19]. Similarly, three out of ten dimensions of two instruments are related to sexual history, sexually-transmitted infections, and sexual violence [15, 16]. Of course, items on sexual relationships in the WSW-RHQ are different from sexuality-related items in those instruments in that WSW-RHQ sexuality-related items pertain to sexual satisfaction and the effects of shift work on sexual relationships.

Menstruation was the fourth dimension of WSW-RHQ. This dimension explained 5.91% of the total variance and included four items on dysmenorrhea, premenstrual syndrome, and menstrual irregularities among women shift workers. Women are very sensitive to their menstruation and consider it as a component of their reproductive health [43]. Some scholars considered menstrual symptoms as good parameters for assessing the effects of occupation on reproductive health [44, 45] and even some of them assessed reproductive health among shift workers using menstrual parameters [17]. Moreover, some studies on women used items on menstruation to evaluate the effects of reproductive health promotion interventions [46] or to assess knowledge and attitude about reproductive health [47].

The fifth dimension of WSW-RHQ was delivery which explained 5.67% of the total variance. The three items of this dimension were related to delivery outcomes among women shift workers. The lowest number of items in this dimension compared with the other dimensions may be due to the limited effects of shift work on delivery outcomes among women shift workers. Like WSW-RHQ, other reproductive health measurement instruments contain items on delivery [15, 16]. The World Health Organization also considers safe delivery as a main component of reproductive health.

Our findings revealed significant correlations between items 15 and 16, 19 and 20, 25 and 26, 27 and 28, and 34 and 35. Measurement error happens when items have not accurately been determined or have not directly been measured. It can also happen due to conceptual similarities between two items or words [33]. Each of these pairs of items conveys an almost similar meaning/concept and hence, significant correlations between the measurement errors of their items are justifiable.

Convergent and discriminant validity assessments in the present study showed that all dimensions had acceptable convergent and discriminant validity. Convergent validity is confirmed when the items of the intended construct are close to each other and share a great proportion of variance, while discriminant validity exists when the items of the construct or its extracted factors are distinct from each other [48].

Cronbach’s alpha, McDonald omega, and inter-item correlation coefficients of WSW-RHQ and all its dimensions showed the acceptable internal consistency of the questionnaire. Moreover, test-retest ICC values showed that the questionnaire has acceptable stability. These findings denote that the items of WSW-RHQ measure a single construct.

### Limitations and strengths

This study had three limitations. First, some WSW-RHQ items were related to events in the past and hence, their assessment might have been associated with recall bias. Second, women shift workers who participated in the study might have had different viewpoints from those who refused participation. Third, sociocultural differences among participants might have affected study findings. The strengths of the study were simplicity of the WSW-RHQ items and short amount of time needed for their answering.

## Conclusion

WSW-RHQ has an acceptable factor structure and internal consistency. It is a valid and reliable instrument for the assessment of reproductive health among women shift workers.

### Implications for clinical practice

The WSW-RHQ can be used in healthcare settings for the assessment of women shift workers’ reproductive health. The results of such assessment would help promote reproductive health among these women.

## Data Availability

The data are available from the corresponding author on reasonable request.

## Abbreviations

WSW-RHQ: Women Shift Workers’ Reproductive Health Questionnaire
RMSEA: Root mean score error of approximation
CFI: Comparative fit index
PCFI: Parsimony comparative fit index
GFI: Goodness of fit index,
AGFI: Adjusted goodness of fit index
CMIN/DF: Minimum discrepancy function divided
NFI: Degrees of freedom, normed fit index
PNFI: Parsimony normal fit index.
